# *miR-381* suppresses C/EBPα-dependent Cx43 expression in breast cancer cells

**DOI:** 10.1042/BSR20150167

**Published:** 2015-11-13

**Authors:** Jia Ming, Yan Zhou, Junze Du, Shenghao Fan, Beibei Pan, Yinhuan Wang, Lingjun Fan, Jun Jiang

**Affiliations:** * Department of Breast, Thyroid and Pancreas Surgery, the Second Affiliated Hospital of Chongqing Medical University, Chongqing, 400010, China; † Breast Disease Center, the First Affiliated Hospital of The Third Military Medical University, Chongqing, 400038, China

**Keywords:** C/EBPα, Cx43, MCF-7, MDA-MB-231, *miR-381*, 3′-UTR

## Abstract

Cx43 (connexin43) is an enhancer of the metastasis of breast cancer cells. Our previous study identified *miR-381* as an indirect suppressor of *Cx43* gene expression, with the precise mechanism being not understood. In the present study, using a reporter gene assay, we found that *miR-381* suppressed Cx43 gene expression via the promoter region −500/−250. With site-directed gene mutation, we demonstrated that *miR-381* could directly bind with the sequences CACUUGUAU in the 3′-UTR so as to inhibit C/EBPα (CCAAT/enhancer-binding protein α) expression. C/EBPα was further identified as a novel transcription factor by binding to a canonic element (AATTGTC) locating at −459/−453 in the promoter region of the *Cx43* gene. Functionally, we demonstrated that *miR-381* suppressed C/EBPα- and Cx43-dependent migration and invasion of breast cancer cells. Finally, we revealed that decreased levels of *miR-381* as well as increased expression of C/EBPα and Cx43 in the metastatic breast cancer cells and tissues. Therefore we are the first to identify that *miR-381* suppresses C/EBPα-dependent Cx43 expression in breast cancer cells. The *miR-381*–C/EBPα–Cx43 axis might be a useful diagnostic and therapeutic target of metastatic breast cancer.

## Abstract (precis)

*miR-381* suppressed CX43 expression by directly targeting the 3′-UTR of C/EBPα, a novel transcription factor of Cx43 in human breast cancer cells. The *miR-381*–Cx43 axis might be a useful diagnostic and therapeutic target of metastatic breast cancer.

## INTRODUCTION

More than 1 million women are diagnosed with breast cancer every year worldwide, accounting for one-tenth of all new cancers and 23% of all female cancer cases [1,2]. Approximately 232340 new cases of invasive breast cancer and 39620 breast cancer deaths are expected to occur among U.S. women in 2013 [2]. One in eight women in the U.S.A. develop breast cancer in their lifetime [2]. The relapse and metastasis are the major causes of this disease-related death [3,4]. Emerging studies focus on the metastatic mechanisms of breast cancer, and the gap junction is suggested to be a major regulator of tumour metastasis [5].

The gap junction, located at the cell membrane, is basically comprised with different Cx (connexin) proteins, which are intimately correlated with diverse cell functions [6,7]. The Cx family include 21 members, among which Cx43 is abundantly expressed in the mammary gland [6]. Cx43 is reported to be a contributor to normal cell migration [8] and tumour cell invasion [9]. We [10] and others [11] also claim that Cx43 induces migration and invasion of breast cancer cells. Regulation of Cx43 expression provides promising strategies in regulating cell functions [11,12].

*Cx43* gene expression is tightly regulated by different transcription factors in transcription level. According to previous studies, Sp1 (specificity protein 1), Sp3, AP-1 (activating protein 1) and c-Jun can bind directly to the promoter region of *Cx43* so as to promote transcription activity [12,13]. In addition, *Cx43* is also intimately regulated by miRNAs at the post-transcription level [10,11,14]. miRNAs are one of the largest groups of post-transcriptional regulators [15]. They are composed with two to eight bases at the 5′-end that could bind to the 3′-UTR of the target genes so as to inhibit mRNA levels and gene expression [15]. By directly or indirectly regulating target gene expression, miRNAs regulate a series of biological processes including cell cycle [16], growth [17], apoptosis [17], differentiation [18] and stress reaction [19]. Emerging evidences indicate that miRNAs are important regulators of metastasis in breast cancer [20,21].

We and others have scanned and identified *miR-1*, *miR-206*, *miR-200a*, *miR-381*, *miR-23a*/*b* and *miR-186* as potent suppressors of *Cx43* [10,11,22]. However, the mechanism of *miR-381*-reduced Cx43 expression was still not revealed. In the present study, we provide a precise mechanism that *miR-381* suppresses C/EBPα (CCAAT/enhancer-binding protein α)-dependent Cx43 expression. The *miR-381*–C/EBPα–Cx43 axis regulates the migration of breast cancer cells, which might shed light on the diagnosis and therapy of metastatic breast cancer.

## MATERIALS AND METHODS

### Reagents

The mimics and inhibitors for human *miR-381* were synthesized by Shanghai GenePharma, as were the mimic NC (negative control) and anti-NC (inhibitor negative control). The detailed sequences of the mimics, inhibitors and controls were described in a previous study [23].

### Cell lines and culture conditions

MDA-MB-231 is a highly aggressive breast cancer cell line, whereas MCF-7 is a non-aggressive one [24]. Both MDA-MB-231 and MCF-7 cells were purchased from the Shanghai Cell Bank of the Chinese Academy of Sciences. The cells were cultured in high-glucose DMEM (Dulbecco's modified Eagle's medium) (Hyclone) containing 10% (v/v) FBS (Gibco).

### Tissue collection

The study was approved by the Ethics Committee of the First Affiliated Hospital of The Third Military Medical University and the Second Affiliated Hospital of Chongqing Medical University. All patient-derived tissues were obtained with their written informed consent.

We collected 20 cases of primary tissues and 20 cases of pulmonary metastases of breast cancer that were pathologically diagnosed at the First Affiliated Hospital of the Third Military Medical University and the Second Affiliated Hospital of Chongqing Medical University from 1 October 2012 to 1 July 2013. The age range of the patients was 32–67 years (median 53 years), and the tumour diameter ranged from 1.5 cm to 4.7 cm (mean 2.8 cm). The patients did not receive pre-operative neoadjuvant chemotherapy or endocrine therapy. The primary cancer tissues were obtained in a sterile manner by modified radical mastectomy. Two or three pieces of the specimens from pulmonary metastases were obtained from metastatic patients during CT (computed tomography)-guided biopsy.

### Real-time PCR

Total RNAs were extracted from tissues or cells with TRIzol® reagent (Invitrogen) according to the manufacturer's protocol. RNAs were transcribed into cDNAs using Omniscript (Qiagen). Quantitative real-time PCR was performed using the 7900HT Fast Real-time PCR system (Applied Biosystems). The mRNA expression levels were normalized to the expression of GAPDH (glyceraldehyde-3-phosphate dehydrogenase). Reactions were carried out in duplicate by using Applied Biosystems Taqman Gene Expression Assays and Universal PCR Master Mix. The relative expression was calculated by the 2(^−ΔΔ^*^CT^*) method. All primer sequences used for PCR are available on request from J.M.

### Immunoblot assay

Total proteins were extracted with cell lysis solution (P0013, Beyotime). Then, 40 μg of protein was separated using SDS/PAGE (10% gel), electrotransferred on to PVDF membranes (catalogue number VVLP02500, Millipore), and incubated with rabbit anti-human Cx43 antibody (1:1000 dilution, catalogue number BA1727, Boster) or rabbit anti-human C/EBPα antibody (1:1000 dilution, catalogue number sc-61, Santa Cruz Biotechnology) at 4°C overnight. Then, anti-rabbit secondary antibody (1:2500 dilution, catalogue number P0110, Beyotime) was incubated for 1 h in room temperature. After three washes, protein bands were quantified from the membrane by densitometry using the Adobe Photoshop version 7.01 imaging system.

### Transwell migration and invasion (Matrigel) assays

MDA-MB-231 and MCF-7 cells were transfected with the Cx43-overexpression plasmids, C/EBPα-overexpression plasmids and/or miRNAs for 36 h, and then the cells were digested and resuspended at 10^5^ cells/ml.

In migration assay, 200 μl of cell suspension was sucked into each insert of the Transwell (polycarbonate membrane with 8.0-μm pore size; catalogue number 3422, BD Bioscience). After culture for 12 h, the upper inserts were air-dried, fixed with paraformaldehyde for 15 min, and stained with 0.1% Crystal Violet, and five fields of view were randomly selected for counting cells under a microscope (×200 magnification). The cell migration activity was described as the relative cell numbers of the transmitted cells. In invasion assay, the similar inserts covered with Matrigel (2 mg/ml; Becton Dickinson) were used to measure the cell invasion ability. Freshly trypsinized cells were suspended at 10^5^ cells/ml. Aliquots of 200 μl cells suspension were plated in the inserts. The lower companion plate well contained RPMI 1640 medium plus 10% (v/v) FBS. After 48 h, the cells that migrated through the permeable membrane were fixed with paraformaldehyde, stained with haematoxylin and eosin, and finally counted.

### Reporter gene of human *Cx43* promoter or 3′-UTR

To observe whether *miR-381* would inhibit Cx43 expression via its promoter regions or 3′-UTR, the reporter gene of the human *Cx43* promoter or 3′-UTR were constructed. Genomic DNA of MDA-MB-231 cells were extracted using a kit from Qiagen (catalogue number 19060). The sequences harbouring promoter regions (upstream of the transcription start site) from −3000 to +10 bp (−3000/+10) were cloned by PCR (catalogue number KOD-201, Toyobo) with the genomic DNA as the template. Likewise, the *Cx43* promoter regions −1500/+10, −500/+10 and −250/+10 were also cloned. After PCR, all cloned DNA fragments were subjected to gel electrophoresis, gel recycling, enzyme digestion and linked into a reporter gene vector pGC4-basic (Invitrogen).

The wild-type or mutated human *Cx43* 3′-UTR sequences (1731 bp) were subcloned into a pMIR-REPORT vector (Invitrogen) to form different reporter genes. The sequences of all primers used in the PCR are available on request from J.M.

### siRNA-mediated silencing of C/EBP**α** expression

MDA-MB-231 cells were transfected with a scrambled siRNA (siNC, 20 nmol/ml) or human C/EBPα-specific siRNAs (si-C/EBPα-1 and si-C/EBPα-2, 20 nmol/ml) (catalogue number GS1050, Qiagen) for 36 h.

### Molecular cloning experiments

For the cloning of human C/EBPα-overexpression constructs, the total cellular RNAs were extracted from MDA-MB-231 cells. According to the instructions of the PrimeScript First Strand cDNA Synthesis Kit (catalogue number 6110A, TaKaRa Bio), reverse transcription was performed to prepare the cDNA for the coding region or 3′-UTR of human C/EBPα.

The human C/EBPα CDS (coding sequence) exons were subcloned into the pCDNA3.1 vector to construct a human C/EBPα overexpression plasmid pCDNA-C/EBPαΔ, which could not be directly inhibited by miRNAs (such as *miR-381*). The human C/EBPα CDS exons plus 3′-UTR were inserted into the pCDNA3.1 vector to construct a human C/EBPα-overexpression plasmid pCDNA-C/EBPα, which could be inhibited by miRNAs (such as *miR-381*).

### Cell transfection, site-directed reporter gene mutation and reporter gene assay

Site-directed reporter gene mutation, cell transfection and reporter gene assays were performed as described in [25]. The transfection was performed using the protocol of Lipofectamine™ 2000 (catalogue number 12566014, Invitrogen). The potential C/EBPα-binding site (AATTGTC) in the *Cx43* promoter region was mutated to AGCTACC with a MutanBEST Kit (catalogue number 401, TaKaRa Bio). The potential *miR-381*-binding site (CACUUGUAU) in the 3′-UTR of the human *CEBPA* gene was mutated to CAGGAUCAU. The luciferase activities of the cell lysates were evaluated according to the manufacturer's instruction (catalogue number E1910, Promega) and the total protein concentration in each assay was measured as an internal control.

### ChIP assay

A ChIP assay was used to measure *miR-381*-mediated binding between C/EBPα protein and *Cx43* promoter DNA. In brief, the cultured MDA-MB-231 cells were fixed with 1% (w/v) formaldehyde, followed by sonication. The supernatants with equal amounts of protein were immunoprecipitated with 1 μg of anti-mouse C/EBPα antibody or anti-rabbit IgG as control by using the ChIP Kit (catalogue number 17-10460, Millipore) according to the manufacturer's protocol. The immunoprecipitates were analysed by PCR to detect the co-immunoprecipitated DNA containing the functional C/EBPα-binding site. The ChIP primers were designed as: forward, 5′-ATTTTTGAAATCTCTCTCC-3′, reverse, 5′-GTGTGAGTGACCTGTTTGAT-3′. The length of the desired product was 100 bp.

### Statistics

Results are shown as means±S.D. Comparisons were performed by using ANOVA for multiple groups or Student's *t* test for two groups. *P*<0.05 was considered statistically significant. Values in Figures not sharing a common superscript letter differ significantly (*P*<0.05).

## RESULTS

### *miR-381* inhibits Cx43 expression by suppressing its promoter activity

To explore the regulatory role of *miR-381* in Cx43 expression, we determined *miR-381*-regulated Cx43 expression at the promoter, mRNA and protein levels. We demonstrated that *miR-381* transfection in MDA-MB-231 cells dramatically suppressed the mRNA (Figure 1A) and protein (Figure 1B) levels of Cx43. Unexpectedly, *miR-381* treatment could not inhibit the luciferase activity of the miRNA reporter gene, which contained the 3′-UTR of the *Cx43* gene (Figure 1C). These results indicate that *miR-381* might regulate Cx43 expression at the transcriptional level. As expected, we demonstrated that transfection of *miR-381* notably suppressed the promoter activity of the *Cx43* gene in MDA-MB-231 cells (Figure 1D). Thus we concluded that *miR-381* inhibited Cx43 expression via suppressing its promoter activity.

**Figure 1:**
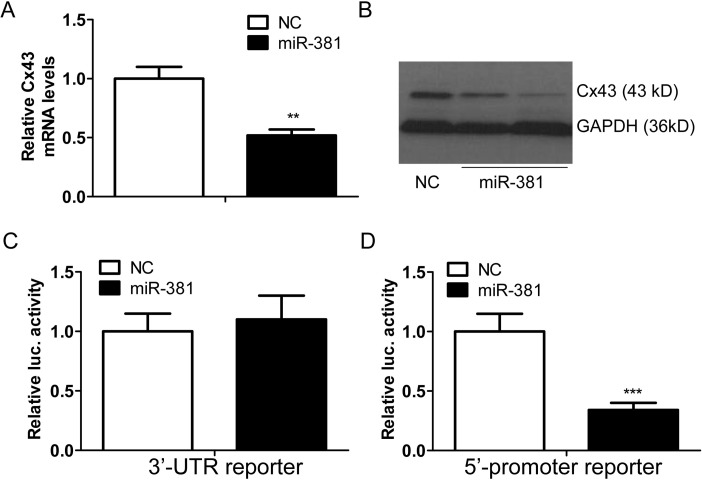
*miR-381* inhibits Cx43 expression by suppressing its promoter activity (**A**) The mRNA levels of Cx43 in MDA-MB-231 cells treated with *miR-381* (100 nM) or scrambled miRNAs (100 nM) as control (NC) for 36 h (*n*=5, **P<0.01). (**B**) Immunoblot assay of Cx43 and GAPDH proteins in MDA-MB-231 cells treated with *miR-381* (100 nM) or NC (100 nM) for 36 h. (**C**) Relative luciferase (Luc.) activity of MDA-MB-231 cells co-transfected with *miR-381* (100 nM) or NC (100 nM) plus a miRNA reporter plasmid (0.4 μg/ml) harbouring the 3′-UTR (1731 bp) of the human *Cx43* gene. (**D**) Relative luciferase (Luc.) activity of MDA-MB-231 cells co-transfected with *miR-381* (100 nM) or NC (100 nM) plus a promoter reporter plasmid (0.4 μg/ml) harbouring the promoter sequences (−3000/+10) of the human *Cx43* gene (*n*=5, ***P<0.005).

### *miR-381* suppresses human Cx43 expression through a potential C/EBPα-binding element in the promoter region of the *Cx43* gene

To determine further the precise mechanism of *miR-381*-reduced promoter activity of *Cx43*, we constructed a series of mutated reporter genes harbouring different lengths (−3000/+10, −1500/+10, −500/+10 and −250/+10) of promoter sequences in the human *Cx43* gene (Figure 2A). Using a reporter gene assay, we found that *miR-381* reduced *Cx43* promoter activity via the −500/−250 region (Figure 2B). Interestingly, by alignment of the human and mouse promoter regions (−3000/0) of *Cx43* with software on the NCBI website (http://blast.ncbi.nlm.nih.gov/Blast.cgi?PAGE_TYPE=BlastSearch&BLAST_SPEC=blast2seq&LINK_LOC=align2seq), two conserved regions, −1064/−935 and −497/−1, in the human *Cx43* gene were discovered (Figure 2C). Consistent with the conserved regions, the −500/−250 sequences might contain some important transcription factor-binding elements. Using online software (http://alggen.lsi.upc.es/), we predicted that two potential binding elements for C/EBPα, AATTGTC located at −459/−453 and TATTGTA located at −324/−318 (Figure 2D and Supplementary Figure 1), might be responsible for *miR-381*-reduced promoter activity of human *Cx43*. To verify this presumption, we performed site-specific gene mutation of these elements, and checked their function using a reporter gene assay. Results showed that *miR-381* notably decreased the luciferase activity of PC-1-WT and PC-1-MUT2, whereas PC-1-MUT1 fully rescued this effect (Figure 2E). These results indicate that *miR-381* inhibited *Cx43* promoter activity via the C/EBPα-binding element AATTGTC located at −459/−453 in the promoter region. To verify further the regulatory role of C/EBPα in *miR-381*-reduced Cx43 expression, we overexpressed C/EBPα in MDA-MB-231 cells. We demonstrated that overexpression of C/EBPα without the 3′-UTR could fully rescue *miR-381*-inhibited Cx43 expression activity, but overexpression of that with 3′-UTR did not have this effect (Figure 2F).

**Figure 2:**
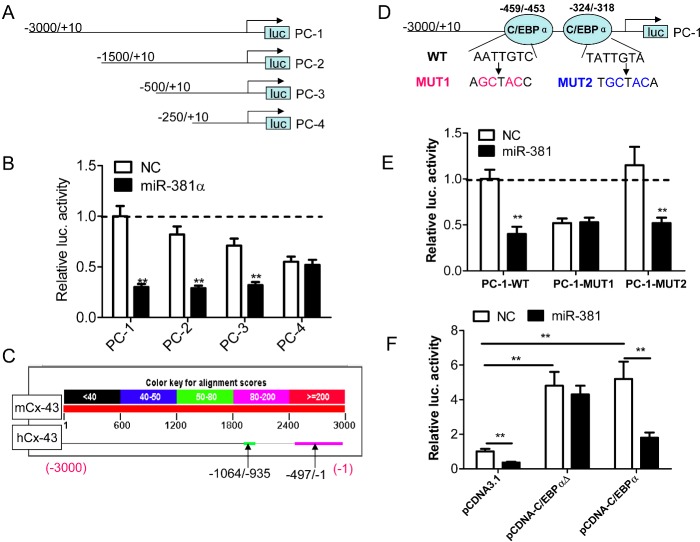
*miR-381* suppresses human Cx43 expression through a potential C/EBPα-binding element in the promoter region of the *Cx43* gene (**A**) The promoter region from −3000 to +10 bp (−3000/+10) in the human *Cx43* gene was subcloned into the pGL4-basic vector to construct a reporter gene PC-1. Likewise, the regions −1500/+10, −500/+10 and −250/+10 were subcloned to construct the reporter genes PC-2, PC-3 and PC-4 respectively. (**B**) Reporter gene assay of PC-1 (0.4 μg/ml), PC-2 (0.4 μg/ml), PC-3 (0.4 μg/ml) or PC-4 (0.4 μg/ml) co-transfected with NC (100 nM) or *miR-381* (100 nM) for 36 h in MDA-MB-231 cells (*n*=4, **P<0.01). (**C**) Alignment of the human and mouse promoter regions (−3000/0) of *Cx43* using the online software on the NCBI website. Two conserved regions (−1064/−935 and −497/−1 in the human *Cx43* gene) were displayed. mCx43, mouse Cx43; hCx43, human Cx43. (**D**) Two potential binding elements of C/EBPα located at −459/−453 (AATTGTC) and −324/−318 (TATTGTA) in the reporter gene PC-1 (WT) were mutated to form the mutated reporter genes PC-1-MUT1 and PC-MUT2 respectively. (**E**) Relative luciferase (Luc.) activity of MDA-MB-231 cells co-transfected with *miR-381* (100 nM) or NC (100 nM) plus PC-1-WT (0.4 μg/ml), PC-1-MUT1 (0.4 μg/ml) or PC-1-MUT2 (0.4 μg/ml) for 36 h. (*n*=5, **P<0.01). (**F**) *miR-381* inhibited human *Cx43* promoter activity via the 3′-UTR of human *CEBPA*. The pCDNA3.1 (0.4 μg/ml), pCDNA-C/EBPαΔ (0.4 μg/ml) or pCDNA-C/EBPα (0.4 μg/ml)-transfected MDA-MB-231 cells were co-transfected with *miR-381* (100 nM) or NC (100 nM) plus the promoter reporter plasmid PC-1 (0.4 μg/ml) for 36 h. Then, the relative luciferase (Luc.) activity was measured. (*n*=5, **P<0.01). pCDNA3.1, empty expression plasmid; pCDNA-C/EBPαΔ, overexpression plasmid harbouring the human *CEBPA* mRNA lacking the 3′-UTR; pCDNA-C/EBPα, an overexpression plasmid containing the human *CEBPA* mRNA containing the 3′-UTR.

### C/EBPα up-regulates Cx43 expression via directly binding to the promoter region of *Cx43*

To verify further that Cx43 is a transcriptional target of C/EBPα, we demonstrated that overexpression of C/EBPα dramatically induced the mRNA (Figure 3A) and protein (Figure 3C) levels of Cx43 in MCF-7 cells. Likewise, silence of C/EBPα by siRNAs notably decreased the mRNA (Figure 3B) and protein (Figure 3D) levels of Cx43 in MDA-MB-231 cells. To determine the direct binding effect between C/EBPα and *Cx43* promoter DNA *in vivo*, a ChIP assay was performed. The results showed that overexpression of C/EBPα increased the binding activity between C/EBPα and *Cx43* promoter DNA (Figure 3E). In addition, siRNA-mediated silence of C/EBPα obtained identical results (Figure 3E).

**Figure 3:**
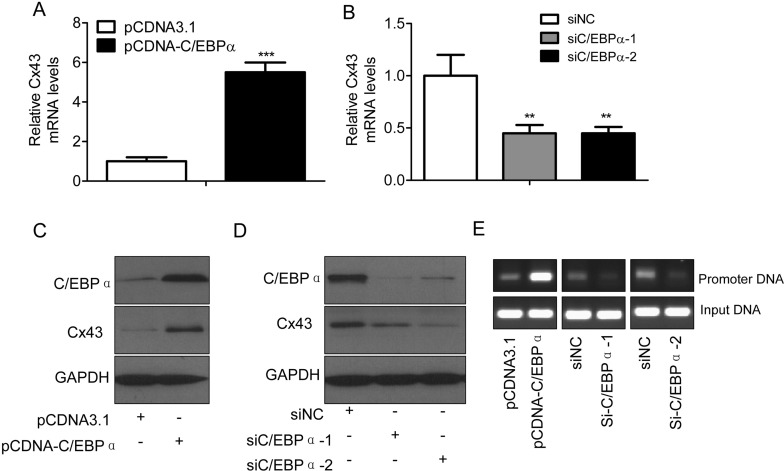
C/EBPα up-regulates Cx43 expression via directly binding to the promoter region of *Cx43* (**A**) Relative mRNA levels of *Cx43* in MCF-7 cells transfected with pCDNA3.1 (0.4 μg/ml) or pCDNA-C/EBPα (0.4 μg/ml) for 36 h (*n*=5, ***P<0.005). (**B**) Relative mRNA levels of *Cx43* in MDA-MB-231 cells transfected with a scrambled siRNA as control (siNC, 20 nmol/ml) or human C/EBPα-specific siRNAs (siC/EBPα-1 or siC/EBPα-2, 20 nmol/ml) for 36 h (*n*=5, **P<0.01). (**C**) Immunoblot assay of Cx43 and C/EBPα in MCF-7 cells transfected with pCDNA3.1 (0.4 μg/ml) or pCDNA-C/EBPα (0.4 μg/ml) for 36 h. (**D**) Immunoblot assay of Cx43 and C/EBPα in MDA-MB-231 cells transfected with siNC (20 nmol/ml), siC/EBPα-1 (20 nmol/ml) or siC/EBPα-2 (20 nmol/ml) for 36 h. (**E**) ChIP assay of MDA-MB-231 cells transfected with siNC (20 nmol/ml), siC/EBPα-1 (20 nmol/ml) or siC/EBPα-2 (20 nmol/ml) for 36 h. The binding activity between C/EBPα protein and *Cx43* promoter DNA was tested.

### *miR-381* suppresses C/EBP**α** expression via directly binding to a consensus site in the 3′-UTR of the *CEBPA* gene

Aforementioned results revealed that *miR-381* suppressed C/EBPα-dependent Cx43 expression in breast cancer cells, whereas the mechanism of *miR-381*-regulated C/EBPα expression was still obscure. We found that *miR-381* treatment significantly decreased the mRNA (Figure 4A) and protein (Figure 4B) levels of C/EBPα in MDA-MB-231 cells. Using online software (http://www.microrna.org/microrna/getMirnaForm.do), we predicted that C/EBPα might be a direct target of *miR-381* (Figure 4C). A potential binding element of *miR-381* in the 3′-UTR of human *CEBPA* mRNA was mutated from CACUUGUAU (WT) to CAGGAUCAU (MUT) to form the miRNA reporter genes (Figure 4C). The reporter gene assay indicated that mutations of the potential binding element could fully rescue *miR-381*-reduced C/EBPα expression activity (Figure 4D). Likewise, *miR-381* transfection significantly attenuated the binding activity between C/EBPα and *Cx43* promoter DNA, whereas this effect was fully rescued by overexpression of an exogenous C/EBPα without the 3′-UTR in MDA-MB-231 cells (Figure 4E).

**Figure 4:**
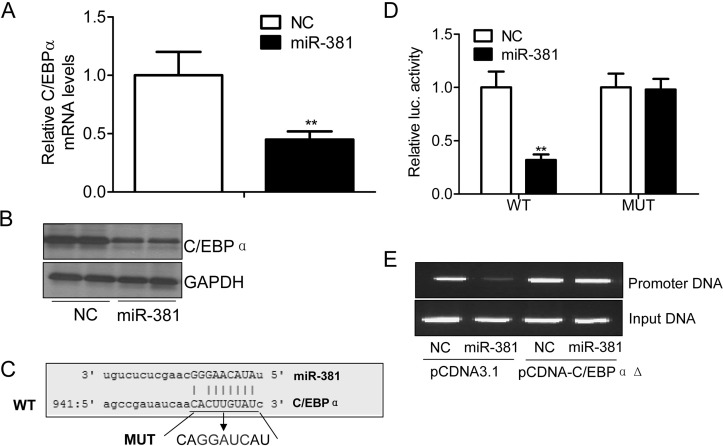
*miR-381* suppresses C/EBPα expression via binding directly to a consensus sites in the 3′-UTR of the *CEBPA* gene (**A**) Relative mRNA levels of *CEBPA* in MDA-MB-231 cells transfected with NC (100 nM) or *miR-381* (100 nM) for 36 h (*n*=5, **P<0.01). (**B**) Immunoblot assay of C/EBPα in MDA-MB-231 cells transfected with NC (100 nM) or *miR-381* (100 nM) for 36 h. (**C**) A potential binding element of *miR-381* in the 3′-UTR of *CEBPA* was mutated to obtain a mutated miRNA reporter gene (MUT). (**D**) The wild-type (WT, 0.4 μg/ml) or the mutated (MUT, 0.4 μg/ml) miRNA reporter genes of human *CEBPA* plus the NC (100 nM) or *miR-381* (100 nM) were co-transfected into MDA-MB-231 cells for 36 h, and then the cells were harvested for luciferase (Luc.) activity assay (*n*=5, **P<0.01). (**E**) ChIP assay of MDA-MB-231 cells transfected with NC (100 nM) or *miR-381* (100 nM) for 36 h. The binding activity between C/EBPα protein and *Cx43* promoter DNA was tested.

### *miR-381* inhibits C/EBPα/Cx43-dependent migration and invasion activity of breast cancer cells

Cx43 was previously identified as a regulator of cell migration [8] and invasion [9]. Thus we presumed that *miR-381*-regulated Cx43 expression would also affect the migration and invasion activity of breast cancer cells. A previous study revealed that MDA-MB-231 cells were highly aggressive, whereas MCF-7 cells were non-aggressive [24]. We found that *miR-381* treatment notably inhibited the migration and invasion activity of MDA-MB-231 cells, and overexpression of Cx43 could fully rescue this effect (Figures 5A and 5B, and Supplementary Figures S2A and S2B). As expected, C/EBPα overexpression produced identical results as Cx43 (Figures 5C and 5D, and Supplementary Figures 2C and 2D). We also confirmed that blockage of *miR-381* in MCF-7 cells robustly induced cell migration and invasion, whereas siRNA-mediated silence of C/EBPα prevented this effect (Figures 5E and 5F, and Supplementary Figures 2E and 2F).

**Figure 5:**
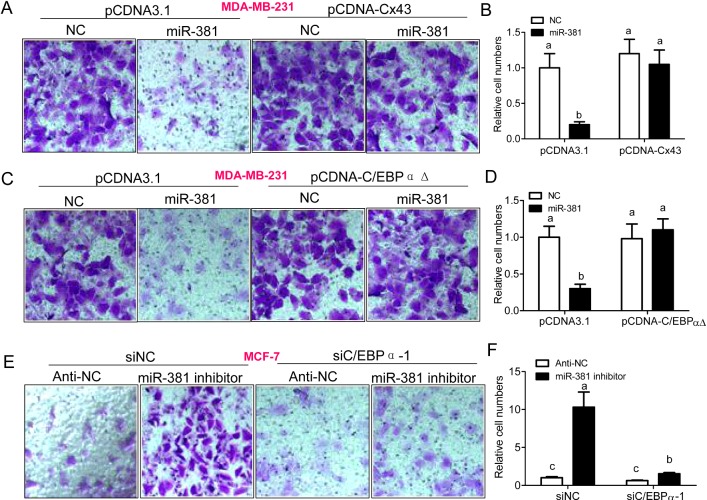
*miR-381* inhibits C/EBPα/Cx43-dependent migration activity of breast cancer cells (**A**) Transwell assay of MDA-MB-231 cells transfected with pCDNA3.1 (0.4 μg/ml) or pCDNA-Cx43 (0.4 μg/ml) plus NC (100 nM) or *miR-381* (100 nM) for 36 h. Representative results are displayed. (**B**) Analysis of the results in (**A**). Values not sharing a common superscript letter differ significantly (*n*=5, *P*<0.05). (**C**) Transwell assay of MDA-MB-231 cells transfected with pCDNA3.1 (0.4 μg/ml) or pCDNA-C/EBPαΔ (0.4 μg/ml) plus NC (100 nM) or *miR-381* (100 nM) for 36 h. Representative results are displayed. (**D**) Analysis of the results in (**C**). Values not sharing a common superscript letter differ significantly (*n*=5, *P*<0.05). (**E**) Transwell assay of MCF-7 cells transfected with siNC (20 nmol/ml) or siC/EBPα-1 (20 nmol/ml) plus NC (100 nM) or *miR-381* inhibitor (100 nM) for 36 h. Representative results are displayed. (**F**) Analysis of the results in (**E**). Values not sharing a common superscript letter differ significantly (*n*=5, *P*<0.05).

### Elevated levels of C/EBPα and Cx43 as well as decreased expression of *miR-381* in aggressive breast cancer cells and tissues

Given the regulatory role of the *miR-381*–C/EBPα–Cx43 axis in the migration activity of breast cancer cells, we further observed the expression of *miR-381*, C/EBPα and Cx43 in aggressive and non-aggressive breast cancer cells and tissues. The aggressive cells MDA-MB-231 expressed much higher levels of Cx43 (Figure 6A) and C/EBPα (Figure 6C), as well as lower levels of *miR-381* (Figure 6B) compared with the non-aggressive MCF-7 cells. Similarly, the metastatic breast cancer tissues expressed much more Cx43 (Figure 6D) and C/EBPα (Figure 6F), as well as much less *miR-381* (Figure 6E) compared with the primary breast cancer tissues.

**Figure 6:**
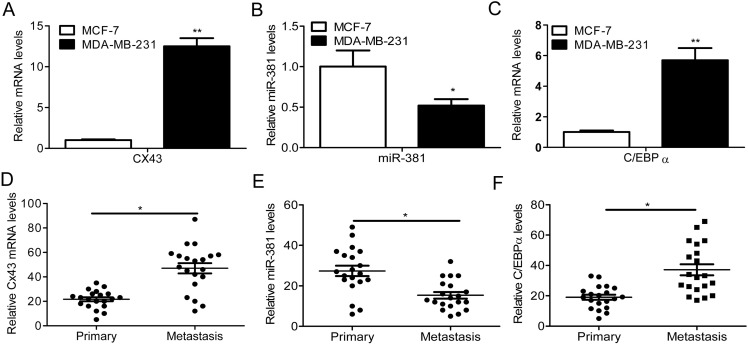
Elevated levels of C/EBPα and Cx43 as well as decreased expression of *miR-381* in aggressive breast cancer cells and tissues (**A**) Relative *Cx43* mRNA levels in MDA-MB-231 cells compared with MCF-7 cells (*n*=5, **P<0.01). (**B**) Relative *miR-381* levels in MDA-MB-231 cells compared with MCF-7 cells (*n*=5, *P<0.05). (**C**) Relative *CEBPA* mRNA levels in MDA-MB-231 cells compared with MCF-7 cells (*n*=5, **P<0.01). (**D**) Relative *Cx43* mRNA levels in primary breast cancer tissues compared with metastatic tissues (*n*=20, *P<0.05). (**E**) Relative *miR-381* levels in primary breast cancer tissues compared with metastatic tissues (*n*=20, *P<0.05). (**F**) Relative *CEBPA* mRNA levels in primary breast cancer tissues compared with metastatic tissues (*n*=20, *P<0.05).

## DISCUSSION

In the present study, we were the first to identify *miR-381* as a suppressor of C/EBPα by binding directly to the 3′-UTR of the *CEBPA* gene. Meanwhile, we also demonstrated that C/EBPα was a novel transcription factor of Cx43. The *miR-381*–C/EBPα–Cx43 axis could regulate the migration and invasion of breast cancer cells (Figure 7).

**Figure 7:**
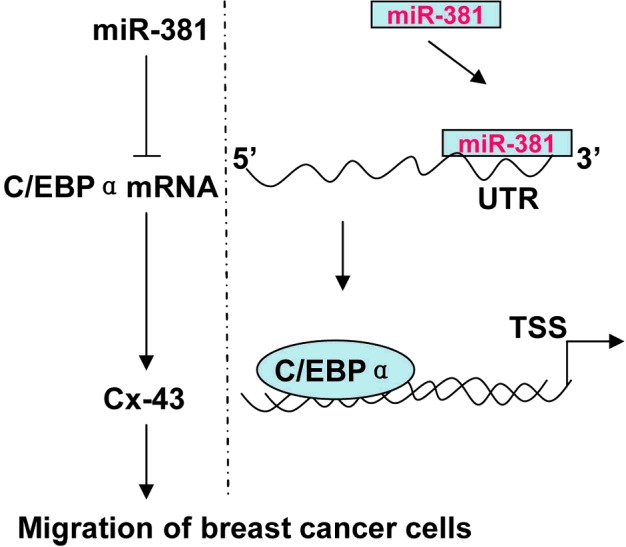
Proposed hypothesis for the *miR-381*–C/EBPα–Cx43 axis in regulating the migration of breast cancer cells *miR-381* inhibited C/EBPα expression by directly binding to the 3′-UTR of the *CEBPA* gene, which was identified as a novel transcription factor of Cx43. The *miR-381*–C/EBPα–Cx43 axis regulates the migration activity of breast cancer cells. The arrow indicates positive and the line indicates negative regulation. TSS, transcription start site.

Cx43 is an important regulator of the metastasis of multiple tumours [9,26–31]. However, whether it exerted a protective or promoting role was still controversial in different cancers. Forster et al. [27] reported that the absence of Cx43 prevented GJIC (gap junctional intercellular communication) and enhanced aggressiveness in pancreatic cancer [27]. Yu et al. [31] suggested that Cx43 sensitized the chemotherapy of non-small-cell lung cancer by inhibiting epithelial–mesenchymal transition. These studies supported the promoting role of Cx43 in tumour metastasis. In contrast, Ghosh et al. [26] reported that Cx43 regulated p38-mediated cell migration and invasion in tumour cells by low doses of γ-radiation in an ERK1/2 (extracellular-signal-regulated kinase 1/2)-independent manner. Likewise, another study suggested that the abnormal expression of Cx43 played an essential role in peritoneal metastasis and that Cx43-mediated heterocellular GJIC between gastric cancer cells and mesothelial cells might be an important regulatory step during metastasis [30]. Therefore these studies supported the promoting role of Cx43 in tumour metastasis. For breast cancer, we were the first to demonstrated that Cx43 might be an enhancer of metastasis by providing several pieces of preliminary evidence: (i) Cx43 was highly expressed in aggressive breast cancer cells and tissues compared with the non-aggressive cancer cells; (ii) overexpression of Cx43 potentiated migration and invasion of breast cancer cells; and (iii) suppressing Cx43 expression by *miR-381* largely inhibited the migration activity of breast cancer cells. It should therefore be pointed out that the diagnostic and therapeutic strategies based on Cx43 need to be designed individually in each specific type of tumour.

The roles of *miR-381* were also controversial in multiple tumours and the related reports were rather limited [32,33]. In line with our findings in the present study, a previous study demonstrated that down-regulation of *miR-381* contributed to the metastatic potential of lung adenocarcinomas via targeting the inhibitor of differentiation 1 [32]. However, Tang et al. [33] suggested that *miR-381* was an ‘oncomir’ in the glioma through suppressing the glioma suppressor leucine-rich repeat C4. These studies indicate that *miR-381* had diverse and complex functions in tumour biology, since *miR-381* might have different targets in different tumours. Whether the role of the *miR-381*–Cx43 axis identified in breast cancer cells in the present study is compatible with other tumours needs to be explored further.

As a transcription factor, C/EBPα could translocate into the nucleus and regulate further a variety of genes directly or indirectly, and have involvement in the regulation of cell differentiation [34,35], proliferation [36] and apoptosis [37]. With regard to cancer research, C/EBPα was suggested to be a tumour suppressor, especially in acute myeloid leukaemia [35]. However, C/EBPα could also be a predictor of poor prognosis in hepatocellular carcinoma [38]. These studies uncovered the contradictory role of C/EBPα in cancer biology. In the present study, by directly targeting Cx43, C/EBPα functioned as a suppressor of metastasis of breast cancer cells. These studies indicate that the role of C/EBPα relies on the special environment of tumours.

The expression activity of Cx43 was regulated at multiple levels, including transcription [13,39], post-transcription [11] and protein phosphorylation [40]. Multiple transcription factors and miRNAs were identified as potent regulators of Cx43 in different cell types. The complex regulatory network of Cx43 expression resulted in its diverse and contradictory functions. More work needs to be performed to verify the unique role of Cx43 in a specific type of tumour.

In conclusion, we have demonstrated that *miR-381* suppressed Cx43 expression by directly targeting the 3′-UTR of *CEBPA*, a novel transcription factor of Cx43 in human breast cancer cells. The *miR-381*–Cx43 axis might be a useful diagnostic and therapeutic target of metastatic breast cancer.

## Abbreviations

CDS: coding sequence
C/EBPα: CCAAT/enhancer-binding protein α
Cx: connexin
GAPDH: glyceraldehyde-3-phosphate dehydrogenase
GJIC: gap junctional intercellular communication
NC: negative control
Sp: specificity protein

## References

[1] Parkin D.M., Bray F., Ferlay J., Pisani P. (2005). Global cancer statistics, 2002. CA Cancer J. Clin..

[2] DeSantis C., Ma J., Bryan L., Jemal A. (2014). Breast cancer statistics, 2013. CA Cancer J. Clin..

[3] Coughlin S.S., Ekwueme D.U. (2009). Breast cancer as a global health concern. Cancer Epidemiol..

[4] Jemal A., Center M.M., DeSantis C., Ward E.M. (2010). Global patterns of cancer incidence and mortality rates and trends. Cancer Epidemiol. Biomarkers Prev..

[5] Defamie N., Chepied A., Mesnil M. (2014). Connexins, gap junctions and tissue invasion. FEBS Lett..

[6] McLachlan E., Shao Q., Laird D.W. (2007). Connexins and gap junctions in mammary gland development and breast cancer progression. J. Membr. Biol..

[7] Smyth J.W., Shaw R.M. (2013). Autoregulation of connexin43 gap junction formation by internally translated isoforms. Cell Rep..

[8] Vliagoftis H., Ebeling C., Ilarraza R., Mahmudi-Azer S., Abel M., Adamko D., Befus A.D., Moqbel R. (2014). Connexin 43 expression on peripheral blood eosinophils: role of gap junctions in transendothelial migration. Biomed. Res. Int..

[9] Ryszawy D., Sarna M., Rak M., Szpak K., Kedracka-Krok S., Michalik M., Siedlar M., Zuba-Surma E., Burda K., Korohoda W. (2014). Functional links between Snail-1 and Cx43 account for the recruitment of Cx43-positive cells into the invasive front of prostate cancer. Carcinogenesis.

[10] Ming J., Zhou Y., Du J., Fan S., Pan B., Wang Y., Fan L., Jun J. (2015). Identification of *miR-200a* as a novel suppressor of connexin-43 in breast cancer cells. Biosci. Rep..

[11] Fu Y., Jiang B.Q., Wu Y., Li Z.D., Zhuang Z.G. (2013). [Hsa-miR-206 inhibits the migration and invasion of breast cancer by targeting Cx43]. Zhonghua Yi Xue Za Zhi.

[12] Villares G.J., Dobroff A.S., Wang H., Zigler M., Melnikova V.O., Huang L., Bar-Eli M. (2009). Overexpression of protease-activated receptor-1 contributes to melanoma metastasis via regulation of connexin 43. Cancer Res..

[13] Gao F.H., Wang Q., Wu Y.L., Li X., Zhao K.W., Chen G.Q. (2007). c-Jun N-terminal kinase mediates AML1-ETO protein-induced connexin-43 expression. Biochem. Biophys. Res. Commun..

[14] Schmidt K., de Wit C. (2015). Keep calm and carry on: miR-1298 prevents up-regulation of Cx43 and secures a quiescent vascular smooth muscle cell. Cardiovasc. Res..

[15] Fellmann C., Hoffmann T., Sridhar V., Hopfgartner B., Muhar M., Roth M., Lai D.Y., Barbosa I.A., Kwon J.S., Guan Y. (2013). An optimized microRNA backbone for effective single-copy RNAi. Cell Rep..

[16] Ghosh T., Aprea J., Nardelli J., Engel H., Selinger C., Mombereau C., Lemonnier T., Moutkine I., Schwendimann L., Dori M. (2014). MicroRNAs establish robustness and adaptability of a critical gene network to regulate progenitor fate decisions during cortical neurogenesis. Cell Rep..

[17] Pollock A., Bian S., Zhang C., Chen Z., Sun T. (2014). Growth of the developing cerebral cortex is controlled by microRNA-7 through the p53 pathway. Cell Rep..

[18] Benaich N., Woodhouse S., Goldie S.J., Mishra A., Quist S.R., Watt F.M. (2014). Rewiring of an epithelial differentiation factor, miR-203, to inhibit human squamous cell carcinoma metastasis. Cell Rep..

[19] Schober A., Nazari-Jahantigh M., Weber C. (2015). MicroRNA-mediated mechanisms of the cellular stress response in atherosclerosis. Nat. Rev. Cardiol..

[20] Iorio M.V., Ferracin M., Liu C.G., Veronese A., Spizzo R., Sabbioni S., Magri E., Pedriali M., Fabbri M., Campiglio M. (2005). MicroRNA gene expression deregulation in human breast cancer. Cancer Res..

[21] Gee H.E., Camps C., Buffa F.M., Colella S., Sheldon H., Gleadle J.M., Ragoussis J., Harris A.L. (2008). MicroRNA-10b and breast cancer metastasis. Nature.

[22] Anderson C., Catoe H., Werner R. (2006). miR-206 regulates connexin43 expression during skeletal muscle development. Nucleic Acids Res..

[23] Xu Y., Ohms S.J., Li Z., Wang Q., Gong G., Hu Y., Mao Z., Shannon M.F., Fan J.Y. (2013). Changes in the expression of miR-381 and miR-495 are inversely associated with the expression of the *MDR1* gene and development of multi-drug resistance. PLoS One.

[24] Lwin Z.M., Guo C., Salim A., Yip G.W., Chew F.T., Nan J., Thike A.A., Tan P.H., Bay B.H. (2010). Clinicopathological significance of calreticulin in breast invasive ductal carcinoma. Mod. Pathol..

[25] Miao H., Zhang Y., Lu Z., Yu L., Gan L. (2012). FOXO1 increases CCL20 to promote NF-κB-dependent lymphocyte chemotaxis. Mol. Endocrinol..

[26] Ghosh S., Kumar A., Tripathi R.P., Chandna S. (2014). Connexin-43 regulates p38-mediated cell migration and invasion induced selectively in tumour cells by low doses of gamma-radiation in an ERK-1/2-independent manner. Carcinogenesis.

[27] Forster T., Rausch V., Zhang Y., Isayev O., Heilmann K., Schoensiegel F., Liu L., Nessling M., Richter K., Labsch S. (2014). Sulforaphane counteracts aggressiveness of pancreatic cancer driven by dysregulated Cx43-mediated gap junctional intercellular communication. Oncotarget.

[28] Brockmeyer P., Jung K., Perske C., Schliephake H., Hemmerlein B. (2014). Membrane connexin 43 acts as an independent prognostic marker in oral squamous cell carcinoma. Int. J. Oncol..

[29] Sirnes S., Lind G.E., Bruun J., Fykerud T.A., Mesnil M., Lothe R.A., Rivedal E., Kolberg M., Leithe E. (2015). Connexins in colorectal cancer pathogenesis. Int. J. Cancer.

[30] Tang B., Peng Z.H., Yu P.W., Yu G., Qian F., Zeng D.Z., Zhao Y.L., Shi Y., Hao Y.X., Luo H.X. (2013). Aberrant expression of Cx43 is associated with the peritoneal metastasis of gastric cancer and Cx43-mediated gap junction enhances gastric cancer cell diapedesis from peritoneal mesothelium. PLoS One.

[31] Yu M., Zhang C., Li L., Dong S., Zhang N., Tong X. (2014). Cx43 reverses the resistance of A549 lung adenocarcinoma cells to cisplatin by inhibiting EMT. Oncol. Rep..

[32] Rothschild S.I., Tschan M.P., Jaggi R., Fey M.F., Gugger M., Gautschi O. (2012). MicroRNA-381 represses ID1 and is deregulated in lung adenocarcinoma. J. Thorac. Oncol..

[33] Tang H., Liu X., Wang Z., She X., Zeng X., Deng M., Liao Q., Guo X., Wang R., Li X. (2011). Interaction of hsa-miR-381 and glioma suppressor LRRC4 is involved in glioma growth. Brain Res..

[34] Chandrasekaran C., Gordon J.I. (1993). Cell lineage-specific and differentiation-dependent patterns of CCAAT/enhancer binding protein alpha expression in the gut epithelium of normal and transgenic mice. Proc. Natl. Acad. Sci. U.S.A..

[35] Rapino F., Robles E.F., Richter-Larrea J.A., Kallin E.M., Martinez-Climent J.A., Graf T. (2013). C/EBPα induces highly efficient macrophage transdifferentiation of B lymphoma and leukemia cell lines and impairs their tumorigenicity. Cell Rep..

[36] Lu H., Yu Z., Liu S., Cui L., Chen X., Yao R. (2015). CUGBP1 promotes cell proliferation and suppresses apoptosis via down-regulating C/EBPα in human non-small cell lung cancers. Med. Oncol..

[37] Xue M., Li X., Wu W., Zhang S., Wu S., Li Z., Chen W. (2014). Upregulation of long non-coding RNA urothelial carcinoma associated 1 by CCAAT/enhancer binding protein alpha contributes to bladder cancer cell growth and reduced apoptosis. Oncol. Rep..

[38] Lu G.D., Ang Y.H., Zhou J., Tamilarasi J., Yan B., Lim Y.C., Srivastava S., Salto-Tellez M., Hui K.M., Shen H.M. (2015). CCAAT/enhancer binding protein α predicts poorer prognosis and prevents energy starvation-induced cell death in hepatocellular carcinoma. Hepatology.

[39] Elzarrad M.K., Haroon A., Willecke K., Dobrowolski R., Gillespie M.N., Al-Mehdi A.B. (2008). Connexin-43 upregulation in micrometastases and tumor vasculature and its role in tumor cell attachment to pulmonary endothelium. BMC Med..

[40] Granot I., Dekel N. (1994). Phosphorylation and expression of connexin-43 ovarian gap junction protein are regulated by luteinizing hormone. J. Biol. Chem..

